# Evaluating the implementation of a chronic obstructive pulmonary disease management program using the Consolidated Framework for Implementation Research: a case study

**DOI:** 10.1186/s12913-021-06636-5

**Published:** 2021-07-21

**Authors:** Stefan Paciocco, Anita Kothari, Christopher J. Licskai, Madonna Ferrone, Shannon L. Sibbald

**Affiliations:** 1 Health and Rehabilitation Sciences, Western University, London, Canada; 2 School of Health Studies, Faculty of Health Sciences, Western University, London, Canada; 3 Department of Family Medicine, Schulich School of Medicine and Dentistry, Western University, London, Canada; 4 RRT-CRE, Asthma Research Group Inc., London, Canada; 5 School of Health Studies, Faculty of Health Sciences, Department of Family Medicine, Schulich School of Medicine and Dentistry, The Schulich Interfaculty Program in Public Health, Schulich School of Medicine and Dentistry, Western University, London, Canada

**Keywords:** Chronic obstructive pulmonary disease, Implementation science, Implementation evaluation, Consolidated framework for Implementation research, Case study, Chronic disease management, Primary healthcare, Interprofessional teams

## Abstract

**Background:**

Chronic obstructive pulmonary disease (COPD) is a prevalent chronic disease that requires comprehensive approaches to manage; it accounts for a significant portion of Canada’s annual healthcare spending. Interprofessional teams are effective at providing chronic disease management that meets the needs of patients. As part of an ongoing initiative, a COPD management program, the Best Care COPD program was implemented in a primary care setting. The objectives of this research were to determine site-specific factors facilitating or impeding the implementation of a COPD program in a new setting, while evaluating the implementation strategy used.

**Methods:**

A qualitative case study was conducted using interviews, focus groups, document analysis, and site visits. Data were deductively analyzed using the Consolidated Framework for Implementation Research (CFIR) to assess the impact of each of its constructs on Best Care COPD program implementation at this site.

**Results:**

Eleven CFIR constructs were determined to meaningfully affect implementation. Five were identified as the most influential in the implementation process. Cosmopolitanism (partnerships with other organizations), networks and communication (amongst program providers), engaging (key individuals to participate in program implementation), design quality and packaging (of the program), and reflecting and evaluating (throughout the implementation process). A peer-to-peer implementation strategy included training of registered respiratory therapists (RRT) as certified respiratory educators and the establishment of a communication network among RRTs to discuss experiences, collectively solve problems, and connect with the program lead.

**Conclusions:**

This study provides a practical example of the various factors that facilitated the implementation of the Best Care COPD program. It also demonstrates the potential of using a peer-to-peer implementation strategy. Focusing on these factors will be useful for informing the continued spread and success of the Best Care COPD program and future implementation of other chronic care programs.

**Supplementary Information:**

The online version contains supplementary material available at 10.1186/s12913-021-06636-5.

## Background

The prevalence of chronic diseases in Canada has increased dramatically within the last few decades [1, 2]. The number of individuals with chronic obstructive pulmonary disease (COPD) has almost doubled since 2000–2001 [3]. COPD is a debilitating chronic respiratory disease that accounts for the greatest number of chronic illness-related hospital admissions in Canada [4].

The use of team-based primary care has been explored to manage and combat the rise of chronic illnesses [5]. Chronic disease management programs using team-based primary care have been successful at mitigating the negative impacts of chronic diseases such as diabetes [6], chronic kidney disease [7], and congestive heart failure [8]. Using primary care to manage chronic diseases has become a successful part of comprehensive care, resulting in these models becoming a standard for chronic disease management and care around the world [9]. Unfortunately, there is limited literature describing how best to engage primary care in the management of COPD utilizing an integrated disease management approach. Indeed, in Canada and other jurisdictions the management of COPD falls below guideline standards, is reactive, not proactive, and in this way distinct from other conditions such as diabetes, where the obverse is true [10–14].

Canada has a universal health care system delivered under provincial jurisdiction. Ontario, Canada’s most populous province (14.7 million), implemented family health teams (FHTs) as a collaborative primary care model consisting of providers from multiple disciplines including primary care clinicians and allied health professionals [15]. Since their implementation in 2005, FHTs have resulted in improved health outcomes and increased access to interprofessional care for patients in Ontario [16]. For patients with COPD who may struggle to navigate the health system, interprofessional team-based primary care is often a better alternative to emergency department or solo practitioner care [17].

Accessibility limits the impact of FHTs in general, and on patients with COPD specifically, as only approximately 20% of the population in Ontario has access to team care within an FHT [15, 18]. The Best Care COPD program (BCC), the subject of this case study, is an efficacious interprofessional team care program that was developed within the FHT context [10]. The impact of BCC and other chronic disease management programs in primary care is dependent on effective implementation.

In order to effectively implement chronic disease management programs context-specific guidance is needed [19]. The implementation of any program into a new setting requires a rich understanding of local context, analysis of stakeholders, and evaluation of provider, organization, and system factors [1].

Using an evidence-based implementation framework for evaluation ensures research is theoretically grounded [20]. There are a number of available frameworks such as promoting action on research implementation in health services [21], the theoretical domains framework [22], and the consolidated framework for implementation research (CFIR) [23]. CFIR, an amalgamation of 19 different theories [23] considers constructs known to affect implementation (see Table 1 and Additional file 1) [24]. Information from CFIR can be used both prescriptively to facilitate the implementation of a program into specific local contexts or retroactively to evaluate implementation efforts [25]. We chose CFIR to evaluate the implementation of the BCC program.

**Table 1:** CFIR Categories and Definitions [24]

CFIR Category	Definition
Intervention Characteristics	The features of an intervention that might influence implementation. Eight constructs are included in intervention characteristics.
Outer Setting	The features of the external context or environment that might influence implementation. Four constructs are included in outer setting.
Inner Setting	The features of the implementing organization that might influence implementation. Twelve constructs are included in inner setting.
Characteristics of Individuals	Characteristics of individuals who are involved in implementation that might influence the implementation. Five constructs are related to this category.
Process	Strategies or tactics that might influence implementation. Eight constructs are related to implementation process.

In Ontario, a team-based COPD management program (BCC) based in primary care focusing on patient self-management through education, skills training and case management, was spread from the region where it was originally developed and implemented, to a neighbouring region using a peer-to-peer implementation approach. While current literature lacks a clear definition, or common name for peer-to-peer approaches in implementation, in general terms, peer-to-peer approaches involve using peer-led education and peer assessment as a method to support learning about the intervention [26–28]. Using a peer-to-peer approach implementation processes can facilitate buy-in and successful uptake/program implementation [27]. Although more research is needed, preliminary evidence shows peer-to-peer learning as facilitating improved clinical education [29, 30]. The purpose of this research was to explore the implementation of the BCC management program at a new clinical site in a different region, using the CFIR framework. Two research objectives guided this study.

1. Determine the enabling and impeding factors to implementation and spread of an interprofessional team-based primary care model, and
2. Explore the peer-to-peer approach to implementing a team-based primary care model.

Current literature lacks context empirical examples of the implementation of team-based primary chronic care models specifically for patients with COPD [31] as well as examples of using a peer-to-peer approach for implementation. Our research set out to fill this gap.

### Case description

The BCC management program is a model of care consisting of primary care practitioners (physicians and nurse practitioners), nurses, a respirologist, RRT’s with certified respiratory educator training, and health administrators all working together to provide COPD-specific care to patients. This model was created for the purposes of “delivering standardized, high-impact best-practices, within an interdisciplinary care model” ([32] p.6). BCC has demonstrated improved patient outcomes (such as reduced severe exacerbations) and reduced urgent health services use (including emergency department visits) [10]. Best practices in the program include creating action plans, skills training (including inhaler and breathing techniques), how to handle exacerbations, spirometry pre- and post-intervention to measure progress, and medication and exercise prescriptions [33]**.** Program standardization and evaluation was supported by a program specific technology solution that guided every encounter and captured performance and outcome metrics [32]. In Canada, health care providers can obtain a certification as certified respiratory educator (a CRE program recognized by the Canadian Network of Respiratory Care)[34]; in this case, all RRTs providing care within BCC had (or obtained) a certified respiratory educator designation.

An important component of the BCC program is an advisory committee, called the Primacy Care Innovation Collaborative (PCIC). The PCIC focuses on healthcare system innovation within primary care including participating in the development of provincial standards [35], and work to better integrate services within primary care through a ‘medical home’ approach [36]. Specific to BCC, PCIC supported and facilitated the robust evaluation and spread of the program outside the original region [32]. As a proof of concept project to demonstrate the programs ability to spread as well as to support the feasibility of the peer-led implementation approach, BCC was spread into a five-site FHT within Ontario (B-FHT). The unit of analysis in this case was considered the FHT. All individuals and organizations external to this were considered the outer setting.

Peer-to-peer implementation of this program was multi-pronged and began with BCC program leads presenting to healthcare teams and practitioners promoting the program. In this case, after the presentation to B-FHT, the BCC RRT program lead worked directly with the RRT at B-FHT to commence program implementation. This began with training providers (RRTs) through both an internal three-day intensive didactic training BCC training process along with the external CRE training requirement. Peer-training continues as patients are recruited and enrolled into the BCC program and new RRTs shadow existing RRTs (and vice versa as new RRTs take on new patients). Peer implementation is also happening concurrently for executive directors – who can reach out to current executive directors already running the BCC program as well as for physicians, who can call on BCC physicians and/or the specialist physician (respirologist) for support. Research is currently on-going exploring the peer-to-peer implementation process in greater detail.

## Methods

Qualitative case study methodology was selected because it allows an in-depth exploration of a single selected entity or case [37]. Stake’s constructivist case study was chosen specifically because he advocates for the researcher’s active involvement in the case [38]. CFIR was used as a theoretical background to collect and analyze the data. CFIR has been used extensively in implementation and evaluation to become aware of influential factors, facilitate analysis, and organize the findings of an implementation [39].

### Setting and participants

The BCC management program evaluated in this study was implemented within one FHT with five clinical sites in Southwestern Ontario. The FHT included different types of providers (physician, nurses, RRTs) and FHT staff working collaboratively to deliver healthcare and management education to patients. Implementation was evaluated at all sites as a single case, since providers interviewed worked across all five sites.

A convenience sample was used for recruitment with all having specific roles on the FHT. Participants included providers implementing BCC within the FHT (i.e., RRTs), providers referring patients to the BCC management program (primary care providers), and patients enrolled in the program (Table 2). Access to participants was granted through the FHT’s executive director. We relied on providers to assist in patient recruitment; recruitment remained ongoing throughout the course of data collection and analysis.

**Table 2:** Participant Characteristics

Participant Type	Number of Participants
Informed Consented Participants	28
**Providers (*n*** **= 24)**	
Executive Director	1
RRT/Certified Respiratory Educators	2
Clinical Lead/Nurse Practitioner	1
Physician	1
Nurse Practitioner	2
Reception	3
Registered Nurse Practitioner	1
Dietitian	1
Registered Nurse	4
Administration	2
Social Worker	4
Kinesiologist	1
Mental Health Counsellor	1
**Patients (*n*** **= 4)**	

### Data collection

Data were collected from a variety of qualitative sources which collectively contributed to the analysis to ensure that the individual, collective, and documented experiences of participants were obtained. Focus groups were conducted to gather the collective experience of the participants [40]. Provider and patient focus groups were conducted independently during sites visits. Observational field notes were taken during the site visits. The purpose of the site visits and field notes were to allow for substantiation of the data through triangulation, as well as to provide an element of reflexivity [38]. Data collection tools were guided by CFIR [24]; interview and focus group questions were built from CFIR as well as from expert opinion (i.e., those involved in the program delivery and implementation). Questions were considered and subsequently selected by the research team with the main goal of eliciting important information about implementation. All questions were piloted and used in previous research [41]. Final focus group and interview guides are available upon request.

One-on-one phone interviews with additional primary care providers who referred patients to the BCC program were conducted. The goal was to gather additional views about the implementation of the program from individuals working indirectly with the program.

Review of FHT documents such as memorandums of understanding, reports, and data sharing agreements produced contextual data that was primarily used to support analysis. Documents were accessed through the executive director of the site and the PCIC.

Throughout the entire research process, written reflexive notes were created by the researchers to ensure that the interviewer’s thoughts and assumptions could be incorporated during data analysis and interpretation [38].

### Data analysis

To ensure a thorough understanding of the context of the site and data collected, an ongoing deductive coding strategy based on CFIR was used, supported by NVivo. Data were coded by 3 researchers (SP, SLS, SM) into related CFIR constructs and sub-constructs. To acknowledge and account for data that did not directly fit into CFIR more effectively (such as patient experience), inductive coding was also performed. Discrepancies were discussed and if there were multiple agreeable codes, segments of data were double-coded. Data categorization methods were performed as per Stake [38]‘s recommendations: direct interpretation and categorical aggregation. All analysis was discussed by the entire research team including a primary care physician. The key enabling constructs were identified as most important during the data analysis process due to being discussed most frequently by the participants and were determined to have a greater impact on implementation through member checking and research team discussion.

While implementation at the FHT took place over 5 sites, the implementation was viewed and evaluated as a single case. Implementation success was qualitatively assessed, using data collected from the provider and patient participants. Throughout data collection, participants reported a high level of satisfaction with the program implementation and delivery.

Field notes and collected documents were integrated into our analysis iteratively. This was done by deductively coding information related to implementation in a similar manner as described above. This additional data was then used in conjunction with focus group and interview data to identify facilitators and barriers to implementation. Participants received an interim report which was discussed during a focus group. Feedback was incorporated into our results. A member check (method of qualitative data triangulation) was conducted to explore the validity of our findings. Member checking involved returning to the implementation site after initial data collection and analysis for a follow-up visit. This allowed the researchers to confirm their interpretation of the data with the participants as well as ask additional questions.

## Results

In total, three focus groups (2 provider, 1 patient), *n* = 1 phone interview, and *n* = 1 key informant interview were conducted involving a total of 28 participants. *n* = 24 providers and *n* = 4 patients (Table 3). All FHT providers invited to participate took part in the study. Informed consent was obtained from all participants prior to any data collection. Response rate for the patients was unknown due to the recruitment of patients being performed by RRTs.

**Table 3:** Type of Data Collection

Type of Data Collection	Number of Sessions	Number of Participants
Provider Focus Groups	2	23
Patient Focus Groups	1	4
Field Notes	3	6
Physician Interview	1	1
Documents	n/a	4
Key Informant Interview^a^	1	1

^a^The key informant also took part in the provider focus groups

### Factors affecting Implementation

Our results are presented according to the 5 main categories of CFIR, while incorporating results from the patient perspective and peer-to-peer implementation. Quotes are provided to illustrate our findings.

#### Intervention characteristics

##### Design quality and packaging

The design quality and packaging of the program was discussed as a critical factor in the decision to adapt the model and its successful implementation. This included the presence of highly trained team members with experience implementing and delivering the BCC program as part of the peer-to-peer approach. They acted in a hands-on and advisory capacity during and following implementation. They trained individuals on how to execute the program as well as offered continued advice post-implementation.

When [the BCC Program Leads] came in, they knew what the expectations were, they knew what the outcomes would look like. They had that experience, where we were just fishing and hoping we would get the outcomes we were hoping for, but we didn’t really have the experience with that to confidently approach all those physician groups (Provider #3, Provider Focus Group #1)*.*

##### Complexity

Participants believed previous program success translated to a smooth process for B-FHT staff in terms of program implementation. “Right away we were sold … it’s an easy sell because they… drop a program and a person attached to it in your lap. It is zero work” (Provider #2, Key Informant Interview #1). The participants further discussed the low complexity of the implementation. “Once… the patients were being seen, there’s not a lot of other admin, oversight really required. It’s the simplest honestly. So simple… everything just fell into place.” (Provider #2, Provider Focus Group #1). Providers elaborated on how they felt the implementation was done effectively and efficiently. “It seems really simple … it didn’t really disrupt … your everyday (Provider #1). If we could roll out every single program that way, it’d be great” (Provider #2, Provider Focus Group #1).

Providers felt the recruiting of patients into the program was smooth and effective. Patients concurred with this statement, saying: “[I] flowed right through [into the new program]” (Patient Focus Group #1). Even though patient awareness of the transition into the program was low; providers believed this facilitated implementation because it did not disrupt usual patient appointments. Providers appreciated being able to spend more time focusing on patient transition and less on other aspects of implementation.

##### Relative advantage

Providers discussed that prior to implementation of the BCC program, B-FHT had been unsuccessful in their attempts to create their own COPD management program. The relative advantage of the BCC program offered a successful and adaptable solution for their patients.

There [were] challenges. One, that there wasn’t an established program, for [the RRT] to mimic. And two … we are a multi-site organization, and with a 0.5 [full-time equivalent RRT] position it is really hard to establish any programming without a consistence presence. Which … just wasn’t possible (Provider #2, Key Informant Interview #1)*.*

Physician #1 echoed this explaining the advantage of having a comprehensive COPD specific care program in “free (ing) me up to focus on other things during appointments” (Phone Interview #1). Providers agreed this was a clear benefit of the program. The physician was confident in the abilities of the newly educated RRTs to provide COPD-specific care to the patients. As a result, they were able to focus their time on a patient’s other concerns and needs, allowing for more efficient use of time during appointments.

Patients also appreciated the coordinate afforded by the BCC program explaining how they prefer this program to alternatives they have previously experienced. “I’ve got a specialist that I’m not agreeing with and that’s not helping me, I might as well not even go to him. [The RRT in this program] is doing [more for me] than he is” (Patient #4, Patient Focus Group #1). From the patient’s view, the RRT was delivering better care for their COPD than was the specialist working external to the program.

Providers discussed the challenges of adding the program’s new reporting technology on top of existing technology. “They have their own system … I hate adding systems. That was the one thing probably that I really was not happy about … we have an [electronic medical record] (EMR). We’re seeing our patients but will be documenting in [the BCC’s system]” (Provider #2, Key Informant Interview #1).

#### Outer setting

##### Patient needs and resources

Providers discussed the patient needs in the community as one of the reasons B-FHT proceeded with program implementation. “COPD was a problem. And COPD patients are complex, time-consuming, and costly. There’s plenty of patients and ongoing work to keep you busy full-time” (Provider #3, Provider Focus Group #1).

##### Cosmopolitanism

Participants described B-FHT’s cosmopolitanism efforts (i.e., efforts to collaboration with external organizations) to be instrumental in implementing the program. There was a shared agreement amongst providers about how “[the BCC’s] guidance was key for us being successful so quickly” (Provider #3, Provider Focus Group #1). In addition to working with the BCC program team, the B-FHT had the chance to learn from the PCIC and strengthen their coordinated efforts with the local hospital.

We actually built it to [be] part of one [program] to refer hospital discharges... with the COPD diagnosis... to automatically send a message to the RRT saying that, that person was discharged (Provider #5, Provider Focus Group #2)*.*

B-FHT had a pre-existing relationship with the local hospital, which had been responsible for performing diagnostic lung function testing (spirometry). The collaboration was challenged because the BCC program standard was for the RRT to complete the spirometry in the local B-FHT office. Transferring spirometry from the hospital to the B-FHT office was a concern because it meant shifting care away from the hospital. A compromise was reached allowing B-FHT to maintain the relationship with the hospital and respecting the requisite program fidelity.

#### Inner setting

##### Networks and communication

In implementing BCC, a network of RRTs was created to enhance communication and facilitate a peer-to-peer approach. This was discussed as a key factor for implementation success because it facilitated information sharing across a broad context which informed practice. This peer-to-peer approach facilitated the training of providers within B-FHT and supported implementation of the program. “The RRTs have their own network where they communicate with each other” (Provider #6, Provider Focus Group #2). “It’s an opportunity for them to … say what’s working, what’s not working, what are they finding out there in the field. They [also] have a [messaging] group” (Provider #4, Provider Focus Group #2). The peer-to-peer approach strengthened communication between providers within the program. BCC Program leads were seen as a highly valued resource for providers. “[She is] always available if we run into any problems or have questions … [we] just reach out to her directly” (Provider #4, Provider Focus Group #2).

Occasionally, poor communication amongst leadership and providers acted as a barrier to implementation. One provider noted “if [meetings are announced] last minute or we forget… it’s just not going to be priority to move all our other appointments around … (Provider #7, Provider Focus Group #2). This was especially significant when the meetings included training or were meant to connect new providers.

##### Readiness for Implementation - available resources

Resource support was also discussed as a factor for successful implementation. Typically, RRTs were newly hired to support program delivery, however “[B-FHT] (used) their existing [RRT] to deliver [the BCC] model” (Provider #4, Provider Focus Group #2). This was both seen as a facilitator (i.e., using available resources) and as a barrier (i.e., requiring unlearning of existing, possibly hindering practices and habits). With the BCC program, the current RRT role was expanded into a full-time position, making it easier to implement the program in B-FHT’s multi-site clinical setting.

Administrative support including affirmation from senior management, secretarial/scheduling support, and chart audit support from the BCC leads was particularly important during the initial stages of implementation. Without this support, RRTs believed they would have had to spend excessive time doing administrative work rather than focusing on patient care. “If you’re rolling a program like this into a [FHT] office without a lot of allied health, those cold calls … for [a patient’s] first visit might be time-consuming if they didn’t have that support” (Provider #3, Provider Focus Group #1).

However, the providers discussed how data in the primary care EMR, distinct from the program electronic health record, was of poor quality. This hampered the ability for the program RRT to identify high risk individuals. Providers felt “better data in [the EMR] would’ve helped. But that’s not… likely or possible” (Provider #2, Provider Focus Group #1).

#### Characteristics of individuals

##### Self-efficacy

Peer-to-peer implementation allowed providers to learn about the program and its intended implementation first-hand from experienced RRTs. Providers felt this increased their confidence in program delivery. “I really appreciate having people who are experts in COPD care that can give me recommendations. The more knowledge I start to feel comfortable with … in COPD in particular is because of [the RRT]” (Provider #3, Provider Focus Group #1).

Throughout focus group discussion, patients remarked how “you follow what [the RRT] says and [what] the doctor says and … my quality of life is better” (Patient #2, Patient Focus Group #1). This trust built between the providers and patients was important for implementation success. Patients reported they felt empowered to manage their care and talked about sharing that with peers and family members.

##### Knowledge and beliefs about the intervention

Providers and patients valued the program from the start. This buy-in of the program enhanced implementation. Providers and patients highly valued the RRT role and expressed many positive views about the RRTs: “If we could clone [the B-FHT RRT], that’s part of what has … made it so successful for us is that she was able to just come in” (Provider #2, Provider Focus Group #1).

#### Process

##### Engaging

Support from senior leadership was essential during the implementation process. The initial impetus to implement the program stemmed from collaboration between RRTs, however, the executive director of the site fully supported and actively facilitated implementation. Participants reported buy-in from senior leadership as a major facilitator to implementation. In addition, other primary care providers engaged with the program throughout the implementation process by learning about program offerings and supporting patient recruitment into the program.

Initially… we were reminded to refer any of our COPD patients for the [RRTs] to make sure that there was a demand. I would really emphasize the importance of frequent reminders to … everyone who would refer patients to the program, reminding them of what kinds of [patients] they can and should refer (Physician #1, Phone Interview #1)*.*

Providers praised the BCC’s intensive approach to early implementation. They explained how it resulted in buy-in from the start.

That initial, really strong blitz on talking to, providing the education to the physicians, speaking with the physician groups individually, getting the searches ready to go... It seemed like that period was probably short, but intense, and necessary (Provider #3, Provider Focus Group #1)*.*

Providers also reported the peer-to-peer approach enabled rapid implementation the program. Peer-to-peer training was conducted by an RRT and supported by a respirologist. Provider #1 said that she “sat with somebody who’d been doing it for twenty, five, and three years” respectively (Provider Focus Group #1). Provider #2 elaborated: “the training piece was also big … that training then does ensure that there’s a consistency in [program delivery]” (Provider Focus Group #1).

##### Reflecting and evaluating

In an effort to collect data on the implementation and performance of the BCC management program, feedback data was collected by B-FHT and the PCIC. Data was collected using patient satisfaction surveys as well as regular debriefing with stakeholders. This data was then used to facilitate adjustments to the implementation and execution of the program as needed.

When discussing the results of this data during reflection of the implementation process, many participants boasted at its success. “I don’t have a single criticism about the program. I really can’t think of how it could have been done better” (Provider #2, Key Informant Interview #1). “We always look at outcome measures, which are always really positive.” (Provider #3, Provider Focus Group #1). Providers explained that not only is patient satisfaction increasing but “hospital admissions had been decreased” (Provider #1, Provider Focus Group #1). When asked to provide advice to other teams considering implementing the program, a key informant said:

Take advantage of this program it is zero work on your end. They will come in and do everything and they will also return. If you are struggling at any point... having trouble identifying patients or... with physician buy-in, if you’re having process issues, they are happy to return... my only advice actually, is “say yes” (Provider #2, Key Informant Interview #1)*.*

Patients echoed provider’s positive views of the program and focused their conversation on the care they received from the program. Patients reflected on their own care and found value in their improved overall quality of life: “[The RRT] was very thorough … with their explanations of your puffers [and] your medication … [the RRT] gave you [advice]… I find it very good, helpful (Patient Focus Group #1).

## Discussion

This implementation case study was the pilot site to evaluate the opportunity for program spread to multiple sites across multiple regions. All of the CFIR constructs analyzed affected implementation, however five were determined as key enabling constructs (based on the frequency of their occurrence in participant comments and in documents) to consider when implementing a team-based chronic care program such as the BCC program: cosmopolitanism, networks and communication, engaging, design quality and packaging, and reflecting and evaluating.

CFIR constructs acting as barriers to be managed during implementation were also identified. These were: complexity (of the new patient reporting system); communication (between providers and management as well as between providers and specialists); and lack available resources (in this case, lack of quality data in the clinic-based primary care EMR).

In a systematic review, Kadu and Stolee [25] evaluated the implementation of chronic care management models in primary care settings. To do this, they used CFIR constructs to determine facilitators and barriers to implementation. Although within each of the 22 studies included there were many combinations of all 39 CFIR constructs affecting implementation, they identified seven constructs which had a meaningful effect on implementation across the studies included in their review. These were: networks and communication, culture, implementation climate, structural characteristics, engaging, executing, readiness for implementation, and knowledge and beliefs about the intervention [25]. Our analysis approach overlapped the Kadu and Stolee [25] review in three constructs: networks and communication, engaging, and knowledge and beliefs about the intervention. This is not to say the others were not important during the implementation of the BCC program, they simply did not appear frequently in our analysis. Our study shows, as others do, that it is a combination of multiple CFIR constructs which meaningfully affect successful implementation [24]. Although Kadu and Stolee [25] evaluated the implementation of chronic care management models in primary care settings, there were no studies among the 22 included that focused specifically on COPD. This may be one reason why only 3 of Kadu and Stolee [25]‘s primary constructs aligned with our finding. Our study adds to this work by providing insight into the implementation of a COPD-specific management program.

### Key enabling CFIR constructs

#### Cosmopolitanism

The strong cosmopolitan relationship developed between B-FHT and the BCC leadership was supported by the evidence-based characteristic of the program. Participants could easily align cognitively and philosophically on evidence-based treatment standards. It also facilitated networking with external organizations. When implementing a chronic care management program, it is important to first consider collaboration with external organizations [1]. The established networks present with PCIC and other RRT networks gave B-FHT providers opportunities to collaborate and gain access to knowledge from a broad network of providers.

Literature on the implementation of chronic care models states that when a collaborative effort is made with external organizations, implementation and sustainability efforts are more effective [1] and factors such as communication, cohesion, and role primacy increase [42]. This finding is mimicked in recent works by Brown et al. [43] and Huang et al. [44]. In our case study, B-FHT’s partnership with the BCC program team acted as a key facilitator to implementation.

#### Networks and communication

Networks and communication or information and communication as termed in Davy et al. [1] are facilitators within literature that were important in this case study. The BCC program in its initial stages created strong networks and communication which supported program delivery [45]. Enhancing communication among providers and establishing provider-specific networks are key components to facilitating the peer-to-peer implementation [46]. Kadu and Stolee [25] uphold that strong implementation efforts require established internal communication networks. This helps improve long-term sustainability, keep track of patients, and proactively notice gaps in service provision [1]. When information and communication systems are not in place or are insufficient, they can become a significant barrier to implementation [47].

#### Engaging

Engaging champions in implementation efforts is a key factor to success [48]. Champions can help increase provider support through enthusiasm and support [48]. Participants identified champions within the PCIC, B-FHT management, and the RRT as peer leaders during implementation. When leadership is engaged, there is more likely to be support from other providers [1, 25]. Alternatively, if leadership is not engaged, stakeholders may begin to lose interest [48].

#### Design quality and packaging

The excellent design and packaging of the intervention positively influenced B-FHT’s decision to implement and supported the ease of implementation. The BCC structured program coupled with the support from BCC program team, the PCIC and other RRT networks infused quality within the whole implementation. Literature has shown that poor design quality or lack of attention to packaging can be a barrier to implementation [47–49]. Well-designed educational materials, such as those used by BCC, can also facilitating implementation by fostering engagement and increasing clarity [50].

#### Reflecting and evaluating

In our study, evidence-based data, specifically regular reflecting on performance metrics and peer-to-peer feedback methods were used effectively to support implementation. Integrating regular monitoring and evaluation throughout program delivery can support implementation efforts [10]. Regular debriefing with stakeholders to allow for critical reflection and evaluation is also important and should be embedded early on in implementation [1, 38]. Feedback systems that are used to support implementation can also work to support program sustainability [1, 49].

### Other CFIR factors affecting implementation

There were other CFIR constructs found to be supportive of implementation, but not necessarily as impactful as those already discussed. **Complexity:** When stakeholders believe an implementation is simple, the program is more easily implemented into practice [51]; overly complex programs or processes can impede the implementation of chronic care models [1]. In our case, the simplicity of implementation, attributed to the high level of support from the BCC program team, was a facilitator – often supporting other key constructs such as engagement and reflection. **Patient Needs and Resources:** There is consensus in the literature around the importance of considering context in implementation efforts - this should consider factors at multiple levels including patient, provider, team, organization, and community [1, 24, 51–53]. A systematic review by Davy et al. [1] described how implementation is enabled when providers believe their intervention helps their patients, rather than a change for change’s sake. The BCC program in our study addressed a clear and growing need for COPD-specific care in the B-FHT community. **Relative Advantage:** Providers believed the program had a relative advantage to what was currently being offered to patients; this, combined with the perceived need within their community, facilitated implementation. Our results echo the findings of Greenhalgh et al. and others [47, 51] who have explained the importance of programs having a “clear, unambiguous advantage in either effectiveness or cost-effectiveness [as] more easily adopted and implemented” [51 p.594]. **Readiness for Implementation – Available Resources:** There is consensus in the literature that a lack of resources, or a misuse of available resources (ex. time, funding, and space) can hinder implementation [1, 54, 55]. In our study, the addition of a full-time RRT position made a meaningful difference in the overall provision of services. While the addition of resources can support implementation, it is important to ensure any resource is added or appropriate for the context [1]. **Self-Efficacy/Knowledge and Beliefs about the Intervention:** Ensuring providers possess the necessary skills to achieve implementation goals is essential [1]. BCC’s training helped build confidence and empowerment in providers, and the peer-to-peer approach facilitated buy-in to the possibilities of the program (i.e., positive outcomes for patients and providers) [43]. Low self-efficacy and high staff turnover can undermine the implementation process [56, 57]. When providers feel more confident in their scope of practice, they can build trust and support implementation.

### Peer-to-peer implementation

Literature describes peer-led education as a powerful approach to achieving program goals and objectives [26–28]. To our knowledge, using a peer-led approach to implementation has not been directly studied, however our case shows the potential for such an approach. In this case RRTs, as regulated health professionals with specialized COPD training as certified respiratory educators, worked directly with other RRTs in implementation and program delivery. Their professional-efficacy and commitment to program goals was amplified by regular peer-to-peer education and training. The providers mentioned how regular training sessions, along with the program lead’s availability throughout implementation, as essential in the success of program implementation and ultimately the program overall. The creation of the RRT network, which allowed RRTs to share emerging ideas and concerns about the program and their patients throughout and following implementation, was seen as a key component successful implementation. Positive views surrounding the program and the critical role of the RRTs during implementation were shared by all participants echoing the findings of Pfadenhauer et al. [58] who explained how key individuals who can be champions during an implementation can enhance overall program success.

### Limitations

Our small sample size and in depth look at one case meant there was a potential for both social desirability bias and the Dunning-Kreuger effect (the belief that implementation was better than it actually was) [59]. The latter point is mitigated by positive outcomes reported in the seminal randomized control trial from which the spread initiative was launched [10]. Participants all worked together which may have affected their willingness to share experiences; however, we ensured there were multiple opportunities for feedback, including our formal member checking. We acknowledge our results are specific to this case study, however our standardized methodology and evaluation framework support our results can being interpreted in other contexts. Triangulation of data and member checking supported the rigor and trustworthiness of our data. Due to the fact this is a single case study, determination of important factors affecting implementation success was based on in-depth discussion with our research team and research participants; we also relied on inference from factors including the frequency with which particular barriers/facilitators were mentioned. Future research is ongoing examining the variability in implementation success across multiple sites and examining differences in facilitators at successful sites versus barrier’s unsuccessful sites. Even though the findings came from a single case study, our results will be useful in planning spread and implementation efforts of the BCC program at other sites and for others wanting to implement a chronic disease management program.

## Conclusion

This study was conducted to understand the facilitators and barriers that affect the implementation of a chronic care management program for patients with COPD in a primary care context. Our aim was to determine enabling factors of implementation and spread of an interprofessional team-based primary care model to support future spread efforts. The five most influential constructs to implementation according to CFIR were cosmopolitanism, networks and communication, engaging, design quality and packaging, and reflecting and evaluating. Our results align with those from the literature including Kadu and Stolee [25]‘s systematic review using CFIR of factors affecting the implementation of chronic disease management programs. The successful implementation of the BCC program within B-FHT can be attributed to multiple factors. The program’s overall success was well regarded by both providers for its positive outcomes and by patients for the improvement in their COPD-specific care. Overall, CFIR was a suitable determinant framework for conducting our study. It provided a broad and useful set of constructs from which was able to determine factors affecting the implementation of the BCC management program. We also aimed to understand the peer-to-peer approach to implementation. This implementation was understood as vital to assist in communication, engagement, and self-efficacy of providers.

This study provides a practical example of the various factors that facilitate the implementation of the BCC management program. It also demonstrates the potential of using a peer-to-peer implementation strategy. Focusing on these factors will be useful for informing the continued spread and success of the BCC program and future implementation of other chronic care programs.

## Supplementary Information

**Additional file 1.** Consolidated Framework for Implementation Research Constructs. This document provides an organized list including the categories and constructs of the consolidated framework for implementation research, as well as a short definition for each construct.

## Data Availability

The datasets generated and analyzed during the current study are not publicly available in order to maintain the confidentiality of the participants but are available from the corresponding author on reasonable request.

## Abbreviations

COPD: Chronic Obstructive Pulmonary Disease
FHT: Family Health Team
CFIR: Consolidated Framework for Implementation Research
EMR: Electronic Medical Record
RRT: Registered Respiratory Therapist
BCC: Best Care COPD
PCIC: Primary Care Innovation Collaborative
